# Evidence of Inbreeding Depression on Human Height

**DOI:** 10.1371/journal.pgen.1002655

**Published:** 2012-07-19

**Authors:** Ruth McQuillan, Niina Eklund, Nicola Pirastu, Maris Kuningas, Brian P. McEvoy, Tõnu Esko, Tanguy Corre, Gail Davies, Marika Kaakinen, Leo-Pekka Lyytikäinen, Kati Kristiansson, Aki S. Havulinna, Martin Gögele, Veronique Vitart, Albert Tenesa, Yurii Aulchenko, Caroline Hayward, Åsa Johansson, Mladen Boban, Sheila Ulivi, Antonietta Robino, Vesna Boraska, Wilmar Igl, Sarah H. Wild, Lina Zgaga, Najaf Amin, Evropi Theodoratou, Ozren Polašek, Giorgia Girotto, Lorna M. Lopez, Cinzia Sala, Jari Lahti, Tiina Laatikainen, Inga Prokopenko, Mart Kals, Jorma Viikari, Jian Yang, Anneli Pouta, Karol Estrada, Albert Hofman, Nelson Freimer, Nicholas G. Martin, Mika Kähönen, Lili Milani, Markku Heliövaara, Erkki Vartiainen, Katri Räikkönen, Corrado Masciullo, John M. Starr, Andrew A. Hicks, Laura Esposito, Ivana Kolčić, Susan M. Farrington, Ben Oostra, Tatijana Zemunik, Harry Campbell, Mirna Kirin, Marina Pehlic, Flavio Faletra, David Porteous, Giorgio Pistis, Elisabeth Widén, Veikko Salomaa, Seppo Koskinen, Krista Fischer, Terho Lehtimäki, Andrew Heath, Mark I. McCarthy, Fernando Rivadeneira, Grant W. Montgomery, Henning Tiemeier, Anna-Liisa Hartikainen, Pamela A. F. Madden, Pio d'Adamo, Nicholas D. Hastie, Ulf Gyllensten, Alan F. Wright, Cornelia M. van Duijn, Malcolm Dunlop, Igor Rudan, Paolo Gasparini, Peter P. Pramstaller, Ian J. Deary, Daniela Toniolo, Johan G. Eriksson, Antti Jula, Olli T. Raitakari, Andres Metspalu, Markus Perola, Marjo-Riitta Järvelin, André Uitterlinden, Peter M. Visscher, James F. Wilson, on behalf of the ROHgen Consortium

**Affiliations:** 1 Centre for Population Health Sciences, University of Edinburgh, Edinburgh, Scotland, United Kingdom; 2 Institute for Molecular Medicine Finland (FIMM), Helsinki, Finland; 3 Department of Chronic Disease Prevention, National Institute for Health and Welfare, Helsinki, Finland; 4 Institute for Maternal and Child Health, IRCCS “Burlo Garofolo,” University of Trieste, Trieste, Italy; 5 Department of Epidemiology, Erasmus University Medical Center, Rotterdam, The Netherlands; 6 Queensland Institute of Medical Research, Brisbane, Queensland, Australia; 7 Estonian Genome Center, University of Tartu, Tartu, Estonia; 8 Institute of Molecular and Cell Biology, University of Tartu, Tartu, Estonia; 9 Division of Genetics and Cell Biology, San Raffaele Research Institute, Milano, Italy; 10 Department of Psychology, University of Edinburgh, Edinburgh, Scotland, United Kingdom; 11 Biocenter Oulu, University of Oulu, Oulu, Finland; 12 Institute of Health Sciences, University of Oulu, Oulu, Finland; 13 Department of Clinical Chemistry, Tampere University Hospital, Tampere, Finland; 14 Department of Clinical Chemistry, University of Tampere School of Medicine, Tampere, Finland; 15 Centre for Biomedicine, European Academy Bozen/Bolzano (EURAC), Bolzano, Italy; 16 MRC Human Genetics Unit, MRC Institute of Genetics and Molecular Medicine, University of Edinburgh, Edinburgh, Scotland, United Kingdom; 17 The Roslin Institute, University of Edinburgh, Easter Bush, Midlothian, Scotland, United Kingdom; 18 Genetic Epidemiology Unit, Department of Epidemiology, Erasmus MC University Medical Center, Rotterdam, The Netherlands; 19 Uppsala Clinical Research, Uppsala University, Uppsala, Sweden; 20 Department of Pharmacology, Faculty of Medicine, University of Split, Split, Croatia; 21 Institute for Maternal and Child Health, IRCCS “Burlo Garofolo,” Trieste, Italy; 22 Department of Biology, Faculty of Medicine, University of Split, Split, Croatia; 23 Department of Immunology, Genetics and Pathology, SciLifeLab Uppsala, Rudbeck Laboratory, Uppsala University, Uppsala, Sweden; 24 Andrija Stampar School of Public Health, Medical School, University of Zagreb, Zagreb, Croatia; 25 Department of Public Health, Faculty of Medicine, University of Split, Split, Croatia; 26 Centre for Global Health, University of Split, Split, Croatia; 27 Centre for Cognitive Ageing and Cognitive Epidemiology, University of Edinburgh, Edinburgh, Scotland, United Kingdom; 28 Institute of Behavioural Sciences, University of Helsinki, Helsinki, Finland; 29 Oxford Centre for Diabetes, Endocrinology, and Metabolism, University of Oxford, Churchill Hospital, Headington, Oxford, United Kingdom; 30 Wellcome Trust Centre for Human Genetics, University of Oxford, Oxford, United Kingdom; 31 Department of Medicine, Turku University Hospital, Turku, Finland; 32 Department of Medicine, University of Turku, Turku, Finland; 33 National Institute for Health and Welfare, Oulu, Finland; 34 Department of Internal Medicine, Erasmus University Medical Center, Rotterdam, The Netherlands; 35 Netherlands Genomics Initiative (NGI)–sponsored Netherlands Consortium for Healthy Aging (NCHA), Leiden, The Netherlands; 36 Brain Research Institute, University of California Los Angeles, Los Angeles, California, United States of America; 37 UCLA Center for Neurobehavioral Genetics, University of California Los Angeles, Los Angeles, California, United States of America; 38 Semel Institute for Neuroscience and Human Behavior, University of California Los Angeles, Los Angeles, California, United States of America; 39 Department of Clinical Physiology, Tampere University Hospital, Tampere, Finland; 40 Department of Clinical Physiology, University of Tampere School of Medicine, Tampere, Finland; 41 Department of Health and Functional Capacity, National Public Health Institute–Helsinki and Turku, Turku, Finland; 42 Division of Welfare and Health Promotion, National Institute for Health and Welfare, Helsinki, Finland; 43 Geriatric Medicine Unit, University of Edinburgh, Royal Victoria Hospital, Edinburgh, Scotland, United Kingdom; 44 CBM scrl – Genomics, Area Science Park, Basovizza, Trieste, Italy; 45 Colon Cancer Genetics Group, Institute of Genetics and Molecular Medicine, University of Edinburgh, Edinburgh, Scotland, United Kingdom; 46 Genetic Epidemiology Unit, Department of Clinical Genetics, Erasmus MC University Medical Center, Rotterdam, The Netherlands; 47 Medical Genetics Section, The University of Edinburgh Molecular Medicine Centre, Institute of Genetics and Molecular Medicine, Western General Hospital, Edinburgh, Scotland, United Kingdom; 48 Department of Health and Functional Capacity, National Public Health Institute, Helsinki, Finland; 49 Department of Psychiatry, Washington University St. Louis, Missouri, United States of America; 50 Oxford NIHR Biomedical Research Centre, Churchill Hospital, Headington, Oxford, United Kingdom; 51 Department of Psychiatry, Erasmus Medical Center, Rotterdam, The Netherlands; 52 Institute of Clinical Medicine/Obstetrics and Gynecology, University of Oulu, Oulu, Finland; 53 Department of Neurology, General Central Hospital, Bolzano, Italy; 54 Department of Neurology, University of Lübeck, Lübeck, Germany; 55 Institute of Molecular Genetics–CNR, Pavia, Italy; 56 Department of General Practice and Primary Health Care, University of Helsinki, Helsinki, Finland; 57 Unit of General Practice, Helsinki University Central Hospital, Helsinki, Finland; 58 Folkhälsan Research Center, Helsinki, Finland; 59 Research Centre of Applied and Preventive Cardiovascular Medicine, University of Turku, Turku, Finland; 60 Department of Clinical Physiology, Turku University Hospital, Turku, Finland; 61 Department of Epidemiology and Biostatistics, School of Public Health, Imperial College London, United Kingdom; Georgia Institute of Technology, United States of America

## Abstract

Stature is a classical and highly heritable complex trait, with 80%–90% of variation explained by genetic factors. In recent years, genome-wide association studies (GWAS) have successfully identified many common additive variants influencing human height; however, little attention has been given to the potential role of recessive genetic effects. Here, we investigated genome-wide recessive effects by an analysis of inbreeding depression on adult height in over 35,000 people from 21 different population samples. We found a highly significant inverse association between height and genome-wide homozygosity, equivalent to a height reduction of up to 3 cm in the offspring of first cousins compared with the offspring of unrelated individuals, an effect which remained after controlling for the effects of socio-economic status, an important confounder (χ^2^ = 83.89, df = 1; *p* = 5.2×10^−20^). There was, however, a high degree of heterogeneity among populations: whereas the direction of the effect was consistent across most population samples, the effect size differed significantly among populations. It is likely that this reflects true biological heterogeneity: whether or not an effect can be observed will depend on both the variance in homozygosity in the population and the chance inheritance of individual recessive genotypes. These results predict that multiple, rare, recessive variants influence human height. Although this exploratory work focuses on height alone, the methodology developed is generally applicable to heritable quantitative traits (QT), paving the way for an investigation into inbreeding effects, and therefore genetic architecture, on a range of QT of biomedical importance.

## Author Summary

Studies investigating the extent to which genetics influences human characteristics such as height have concentrated mainly on common variants of genes, where having one or two copies of a given variant influences the trait or risk of disease. This study explores whether a different type of genetic variant might also be important. We investigate the role of recessive genetic variants, where two identical copies of a variant are required to have an effect. By measuring genome-wide homozygosity—the phenomenon of inheriting two identical copies at a given point of the genome—in 35,000 individuals from 21 European populations, and by comparing this to individual height, we found that the more homozygous the genome, the shorter the individual. The offspring of first cousins (who have increased homozygosity) were predicted to be up to 3 cm shorter on average than the offspring of unrelated parents. Height is influenced by the combined effect of many recessive variants dispersed across the genome. This may also be true for other human characteristics and diseases, opening up a new way to understand how genetic variation influences our health.

## Introduction

Height is a classic complex trait, which is influenced by both genetic and non-genetic factors. Observed increases in height in developed countries over the last few generations suggest that environmental factors such as nutrition and childhood healthcare play an important role in determining adult height [1], [2]. Within any one population at one point in time, 80–90% of the variation in height is explained by genetic factors [3], [4], [5], [6], [7], [8]. These characteristics, plus the fact that height is cheaply and accurately measurable and has been assessed in many thousands of study subjects, make it an attractive model for investigating the genetic architecture of quantitative traits generally [9], . Height is not merely of interest as a model quantitative trait (QT): a better understanding of the genetic mechanisms influencing height offers insights into genetic variants influencing growth and development [11]. Because height is associated with a range of complex diseases, including cancer, [12], [13], [14], [15] and because pleiotropic effects have been observed between disease-associated and height-associated genetic variants [16], [17], [18], a better understanding of the genetic mechanisms influencing height may also provide biological insights into disease mechanisms.

In a seminal work published almost a century ago, Fisher first proposed that the heritability of height results from the combined effects of many genetic variants of individually small effect size [19]. In recent years, the advent of genome-wide association studies (GWAS) has enabled this theory to be tested empirically. A GWAS of over 180,000 individuals conducted by the GIANT (Genome-wide Investigation of Anthropometric Measures) consortium found common genetic variants at more than 180 loci influencing human height [20]. Despite the undoubted success of GWAS, even this very large study discovered variants explaining in total only around 10% of phenotypic variation [20]. This “missing heritability” [21] has become an important subject of debate in genetic epidemiology because of the implications it has for future gene discovery strategies and indirectly on attempts to predict phenotype from genotype. Yang and colleagues proposed a different approach to identifying this missing heritability [22]. Instead of using GWAS to identify individual genome-wide significant SNPs associated with stature, they considered all SNPs simultaneously, allowing the entire GWAS data to be used as predictors. Using this approach, they explained up to 40% of the variance in height. This still leaves ∼40% of variance unexplained by common genetic variants. The authors of the large GIANT study cited above predict that increased GWAS sample sizes will identify more common variants of moderate-to-small effect size and will increase the proportion of heritable variation explained merely to around 20% [20], [22]). Therefore, alternative strategies are required in order to detect rarer variants, structural variants, variants of very small effect size, and interactions, including dominance and epistasis [21].

This study explores whether there is evidence for genome-wide recessive genetic effects, or inbreeding depression, on height. Inbreeding depression implies directional dominance: i.e. that dominance is on average in the same direction across loci. An association between height and genome-wide homozygosity would imply that height was influenced by the combined effects of many recessive variants of individually small effect size, scattered across the genome. On the face of it, this endeavour looks unpromising. Most pedigree and GWAS studies investigating the genetic architecture of height to date have found no strong evidence of deviation from an additive genetic model [23]. Three heritability studies have found little evidence for dominance variance [24], [25], [26]. Absence of evidence for dominance variance need not, however, be inconsistent with evidence of inbreeding depression: it can be shown that, assuming a large number of contributing loci, it is theoretically possible to have inbreeding depression in the absence of detectable dominance variance [27]. Dominance variance may be difficult to estimate in study designs where genome-wide additive and dominance coefficients are highly correlated [26]. Independently of GWAS, epidemiologists have long observed associations between parental relatedness and reduced height [28], [29], [30], [31], although not all studies have found such an association [32], [33]. A recent small study of the isolated Norfolk Island population found an association between reduced height and both parental relatedness (estimated from genealogical data) and genome-wide homozygosity (estimated from microsatellite markers) [34]. Finally, whilst many twin studies have concluded that height is purely additive, an extended twin family design using large numbers (n = 29,691) revealed a non-additive genetic component of 9.4% which was balanced by extra additive variance due to assortative mating (confounded with shared environment in twin studies). As assortative mating increases the correlation in dizygotic twins above half that in monozygotic twins, whereas dominance does the opposite, they appear to cancel each other out, so height looks perfectly additive from twins alone [35].

The aim of this study was to explore the association between genome-wide homozygosity and adult height, controlling for the effects of potential confounding factors. The study involved over 35,000 subjects, drawn from 21 population samples. We invited studies to participate in the consortium which we knew were conducted in isolated populations, where both the mean and variance in genome-wide homozygosity are higher. In this way, we optimised our chances of being able to detect an effect, should one exist. We found highly significant evidence of an inverse association between genome-wide homozygosity and height, with significant heterogeneity among sample sets.

## Results

We explored the association between genome-wide homozygosity and height in 21 European or European-heritage populations (Table 1). All samples were genotyped using the Illumina platform (see Materials and Methods and Supporting Information). Because different Illumina platforms were used by different studies, we extracted the SNPs present in the Illumina HumanHap 300 panel (common to all the Illumina platforms used). The number of SNPs remaining after quality control procedures had been run on a population-by-population basis are given in Table 1, as are details of the mean age and height of the samples and the proportion of women in each sample.

**Table 1:** Sample details.

Study	Location	N (% female)	Platform^1^	N SNPs after QC	N SNPs in LD pruned panel	Height (cm) mean (SD)	Age (years) mean (SD)
CROATIA-Korčula^3^	Dalmatian Island, Croatia	866 (64)	370	318,448	48,168	168.1 (9.2)	55.8 (13.7)
CROATIA-Split^2^	City of Split, Croatia	499 (43)	370	325,070	33,718	172.5 (9.5)	49.0 (14.7)
CROATIA-Vis^3^	Dalmatian Island, Croatia	778 (59)	300	299,337	47,802	167.8 (10.0)	56.5 (15.3)
EGCUT^2^	National collection, Estonia	2395 (52)	370	321,859	33,852	172.3 (9.7)	40.1 (16.2)
ERF^3^	Village in the Netherlands	789 (62)	300	307,909	43,019	165.0 (8.9)	51.1 (14.2)
FINRISK^2^	Finland	1884 (47)	610	300,312	45,433	169.9 (9.9)	55.7 (12.1)
HBCS^4^	Helsinki, Finland	1721 (57)	610	298,835	45,479	169.0 (8.8)	61.5 (2.9)
H2000^2^	Finland	2101 (51)	610	300,493	45,159	169.6 (9.5)	50.7 (11.1)
INGI-CARL^3^	Village in Italy	430 (62)	370	300,235	48,204	159.8 (9.6)	50.4 (16.3)
INGI-FVG^3^	Villages in Italy	961	370	300,648	47,960	168.7 (9.3)	50.9 (15.6)
INGI-VB^3^	Villages in a valley in Italy	1661 (56)	370	305,451	48,217	164.7 (9.7)	54.7 (18.3)
LBC1921^4^	Lothian Region, Scotland	512 (58)	610	297,795	46,827	163.2 (9.2)	79.1 (0.6)
LBC1936^4^	Lothian Region, Scotland	1005 (49)	610	297,795	47,139	166.5 (8.9)	69.6 (0.8)
MICROS^3^	Villages in a valley in Italy	1079 (57)	300	307,473	47,118	166.2 (9.4)	45.2 (16.1)
NFBC1966^4^	Northern Finland	4988 (52)	370	302,524	44,560	171.2 (9.2)	31.0 (0)
NSPHS^3^	Village in Northern Sweden	638 (53)	300	303,583	34,917	164.3 (9.6)	47.1 (20.7)
ORCADES^3^	Orkney Islands, Scotland	697 (54)	300	306,689	45,208	167.4 (9.4)	55.0 (15.4)
QIMR^2^	NW Europeans, Australia	3925 (58)	370, 610	295,000	31,760	169.2 (9.7)	39.7 (18.0)
RS^2^	Rotterdam, Netherlands	5737 (59)	300	307,042	49,162	166.9 (9.3)	69.0 (8.8)
SOCCS^5^	National collection, Scotland	842 (51)	300	306,310	46,781	169.2 (9.6)	50.7 (5.9)
YFS^2^	Finland	2437 (54)	670	299,112	44,890	172.2 (9.3)	37.7 (5.0)

1 All data were analysed using Illumina SNP arrays. 300 refers to the Illumina HumanHap 300 panel, 370 to the Illumina HumanHap 370 Duo/Quad panels, 610 to the Illumina Human 610 Quad panel and 670 to the Illumina Human 670 Quad panel. In order to harmonise the data, the analysis was conducted using only those SNPs present in the HumanHap 300 panel.

2 Population-based studies.

3 Population-based studies in isolated populations.

4 Birth cohort studies.

5 Case control studies.

We used three different measures of genome-wide homozygosity. F_ROH_ is defined as the percentage of the typed autosomal genome in runs of homozygosity (ROH) greater than or equal to 1.5 Mb in length. F_ROH_ is strongly correlated with the degree of relatedness between an individual's parents [36]. F_ROHLD_ is a modification of F_ROH_, derived using a panel of independent SNPs, where all SNPs in strong linkage disequilibrium (LD) have been removed. This is a more stringent estimate of parental relatedness: removing SNPs that are in strong LD with other SNPs means that all ROH detected are likely to be the result of recent parental relatedness and not ancient patterns of shared ancestry. The third measure we used was observed homozygosity (F_hom_). This is defined as the number of observed homozygous genotypes per individual, expressed as a percentage of the number of non-missing genotypes for that individual. This is a much less precise estimate of parental relatedness, as F_hom_ is a single-point measure which captures all genotyped homozygous loci, not just those located in long ROH. Thus it reflects not only recent parental relatedness but also more ancient aspects of population history, such as population isolation and bottlenecks.

**Figure 1:**
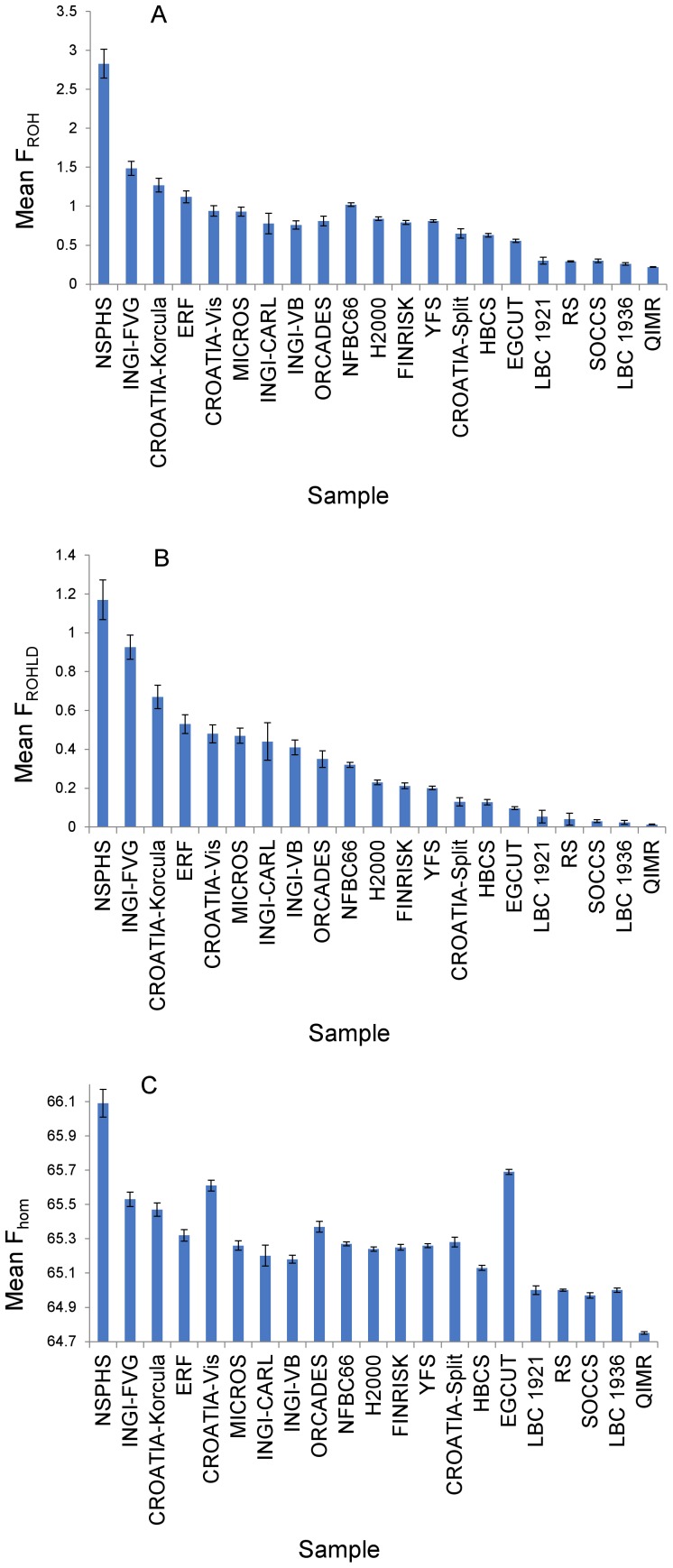
Three alternative measures of mean homozygosity, with 95% confidence intervals, by population sample. (A) shows mean F_ROH_ by population sample. F_ROH_ is defined as the percentage of the genotyped autosomal genome in ROH measuring at least 1.5 Mb. Mean values of F_ROH_ per population (with 95% confidence intervals) are: CROATIA-Korčula = 1.27 (1.18, 1.36); CROATIA-Split = 0.65 (0.59, 0.71); CROATIA-Vis = 0.94 (0.87,1.01); EGCUT = 0.56 (0.54, 0.58); ERF = 1.12 (1.04, 1.20); FINRISK = 0.79 (0.77, 0.82); HBCS = 0.63 (0.60, 0.65); H2000 = 0.84 (0.82, 0.86); INGI-CARL = 0.78 (0.65, 0.91); INGI-FVG = 1.49 (1.40, 1.58); INGI-VB = 0.76 (0.71, 0.81); LBC1921 = 0.30 (0.25, 0.35); LBC1936 = 0.26 (0.24, 0.28); MICROS = 0.93 (0.87, 0.99); NFBC1966 = 1.02 (1.00, 1.04); NSPHS = 2.83 (2.64, 3.02); ORCADES = 0.81 (0.75, 0.87); QIMR = 0.22 (0.21, 0.23); RS = 0.29 (0.28, 0.30); SOCCS = 0.30 (0.28, 0.32); YFS = 0.81 (0.79, 0.83). (B) shows mean F_ROHLD_ by population sample. F_ROHLD_ is defined as the percentage of the genotyped autosomal genome in ROH measuring at least 1.0 Mb, derived from a panel of independent SNPs. Mean values of F_ROHLD_ per population (with 95% confidence intervals) are: CROATIA-Korčula = 0.67 (0.61, 0.73); CROATIA-Split = 0.13 (0.11, 0.15); CROATIA-Vis = 0.48 (0.43, 0.53); EGCUT = 0.10 (0.09, 0.10); ERF = 0.53 (0.48, 0.58); FINRISK = 0.21 (0.20, 0.23); HBCS = 0.13 (0.11, 0.14); H2000 = 0.23 (0.22, 0.24); INGI-CARL = 0.44 (0.34, 0.54); INGI-FVG = 0.93 (0.86, 0.99); INGI-VB = 0.41 (037, 0.45); LBC1921 = 0.05 (0.02, 0.09); LBC1936 = 0.02 (0.01, 0.03); MICROS = 0.47 (0.43, 0.51); NFBC1966 = 0.32 (0.31, 0.33); NSPHS = 1.17 (1.07, 1.27); ORCADES = 0.35 (0.31, 0.39); QIMR = 0.013 (0.011, 0.015); RS = 0.04 (0.01, 0.07); SOCCS = 0.03 (0.02, 0.04); YFS = 0.20 (0.19, 0.21). (C) shows mean F_hom_ by population sample. F_hom_ is defined as the percentage of genotyped autosomal SNPs that are homozygous. Mean values of F_hom_ per population (with 95% confidence intervals) are: CROATIA-Korčula = 65.47 (65.43, 65.51); CROATIA-Split = 65.28 (65.25, 65.31); CROATIA-Vis = 65.61 (65.58, 65.64); EGCUT = 65.69 (65.68, 65.70); ERF = 65.32 (65.29, 65.35); FINRISK = 65.25 (65.23, 65.27); HBCS = 65.13 (65.12, 65.14); H2000 = 65.24 (65.23, 65.25); INGI-CARL = 65.20 (65.14, 65.26); INGI-FVG = 65.53 (65.49, 65.57); INGI-VB = 65.18 (65.16, 65.20); LBC1921 = 65.00 (64.97, 65.03); LBC1936 = 65.00 (64.99, 65.01); MICROS = 65.26 (65.23, 65.29); NFBC1966 = 65.27 (65.26, 65.28); NSPHS = 66.09 (66.01, 66.17); ORCADES = 65.37 (65.34, 65.40); QIMR = 64.75 (64.74, 64.76); RS = 65.00 (64.99, 65.01); SOCCS = 64.97 (64.95, 64.99); YFS = 65.26 (65.25, 65.27).

Figure 1 shows the sample means, with 95% confidence intervals, of these three measures of genome-wide homozygosity. Whereas in general the three measures were strongly correlated, differences were observed, particularly between F_ROHLD_ and F_hom_. For example, the Estonian sample (Estonian Genome Centre of University of Tartu [EGCUT]) had the second highest mean value for F_hom_, but it had one of the lowest mean values for F_ROHLD_. For all three measures of genome-wide homozygosity there is a continuum of values. The isolate populations are generally located at the more homozygous end of the spectrum, but with considerable variation amongst the different sample sets. For example, there is almost a three-fold difference in mean F_ROHLD_ between the Northern Sweden Population Health Study (NSPHS) and ORCADES. The Finnish sample sets and some others (for example, CROATIA-Split and EGCUT) have intermediate levels of homozygosity, whilst the urban and national collections from Scotland, the Netherlands and Australia are the least homozygous. There was more than an order of magnitude difference in mean F_ROHLD_ between the most and the least homozygous population samples.

The purpose of the first part of the analysis was to explore the association between height and homozygosity, as measured in different ways. First, we estimated the association between height and F_ROH_, adjusting for age, sex and (in sample sets including related individuals) genomic kinship (Table 2, Figure S1). We found evidence for a small but strongly significant (*p* = 1.23×10^−11^) inverse association between F_ROH_ and height. This association was significant in nine of the twenty-one sample sets in the study. In nine further sample sets, confidence intervals overlapped with zero but the direction of the effect was consistent with an inverse association between F_ROH_ and height. In none of the sample sets was there a significant positive association between F_ROH_ and height. An increase of 1% in F_ROH_ was associated with a decrease of 0.012 (SE = 0.0018) in the z-score for height (approximately 0.09 cm). Using pedigree and F_ROH_ data from three separate population samples, we estimated that this is equivalent to a reduction in height of 0.7 cm in the offspring of first cousins, compared with the offspring of unrelated individuals (based on F_ROH_ differences of 6.6, 7.4 and 7.4 in the offspring of first cousins compared with the offspring of unrelated individuals in the Micro-Isolates in South Tyrol (MICROS), ORCADES and Irish data sets respectively – see Materials and Methods).

**Table 2:** Meta-analysis of the association between height and genome-wide homozygosity, adjusted for age and sex only.

Homozygosity measure	Number of participants	Effect size (z-score units)	95% Confidence Interval	p-value	p-value (heterogeneity)
**F_ROH_**	35,808	−0.012	−0.015, −0.008	1.23×10^−11^	4.7×10^−16^
**F_ROHLD_**	35,808	−0.065	−0.071, −0.058	1.40×10^−88^	3.7×10^−7^
**F_hom_**	35,378	−0.11	−0.12, −0.10	1.10×10^−83^	8.7×10^−19^
**F_hom_ adj F_ROH_**	35,378	−0.023	−0.030, −0.016	5.36×10^−11^	1.5×10^−124^

**Figure 2:**
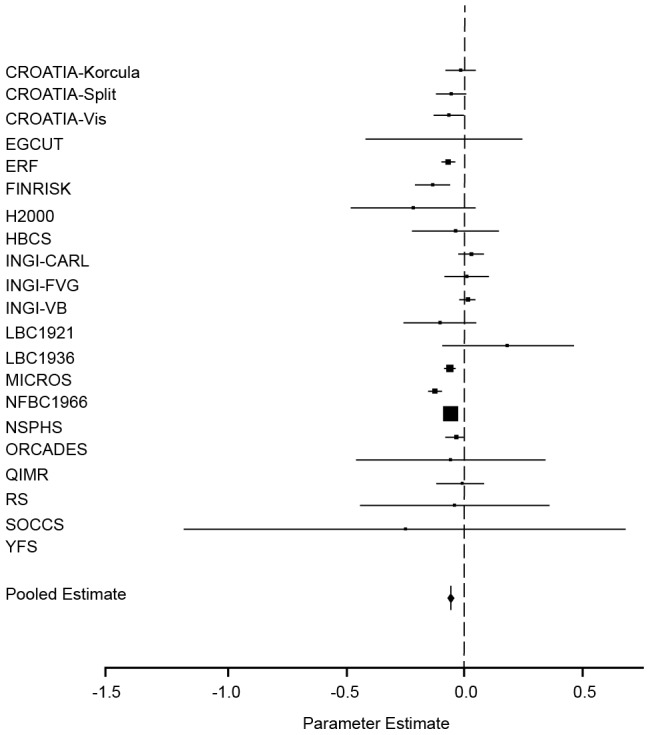
Forest plot of the effect of F_ROHLD_ on height. Results of a meta-analysis of the association between F_ROHLD_ and height are shown for twenty-one population samples. The model was adjusted for age and sex in all samples. Additionally, it was adjusted for genomic kinship in samples with pairs of related individuals (CROATIA-Korčula, CROATIA-Split, CROATIA-Vis, ERF, FINRISK, HBCS, H2000, INGI-CARL, INGI-FVG, INGI-VB, MICROS, NFBC1966, NSPHS, ORCADES and YFS). The plot shows estimated effect sizes (solid squares) for each population, with 95% confidence intervals (horizontal lines). Each sample estimate is weighted by the inverse of the squared standard error of the regression coefficient, so that the smaller the standard error of the study, the greater the contribution it makes to the pooled regression coefficient. The area of the solid squares is proportional to the weighting given to each study in the meta-analysis. Effect sizes in z-score units (with 95% confidence intervals) are: CROATIA-Korčula = −0.02 (−0.09, 0.04); CROATIA-Split = −0.06 (−0.1, −0.002); CROATIA-Vis = −0.07 (−0.1, −0.01); EGCUT = −0.09 (−0.04, 0.2); ERF = −0.08 (−0.1, −0.05); FINRISK = −0.1 (−0.2, −0.07); HBCS = −0.04 (−0.2, 0.1); H2000 = −0.2 (−0.5, 0.04); INGI-CARL = 0.02 (−0.03, 0.07); INGI-FVG = −0.0001 (−0.08, 0.08); INGI-VB = 0.005 (−0.03, 0.04); LBC1921 = −0.1 (−0.3, 0.04); LBC1936 = 0.2 (−0.1, 0.4); MICROS = −0.06 (−0.08, −0.05); NFBC1966 = −0.1 (−0.2, −0.1); NSPHS = −0.07 (−0.07, −0.06); ORCADES = −0.04 (−0.08, 0.001); QIMR = −0.07 (−0.5, 0.3); RS = −0.02 (−0.1, 0.08); SOCCS = −0.05 (−0.4, 0.3); YFS = −0.3 (−1.2, 0.7).

The second analysis estimated the association between height and F_ROHLD_, adjusted for age, sex and genomic kinship. Again, there was evidence of a very strongly significant inverse association (*p* = 1.40×10^−88^) between F_ROHLD_ and height (Table 2, Figure 2). This association was significant in seven of the twenty-one sample sets in this study. In eleven further sample sets, confidence intervals overlapped with zero but the direction of the effect was consistent with an inverse association between F_ROHLD_ and height. In none of the sample sets was there a significant effect in the other direction. A 1% increase in F_ROHLD_ was associated with a decrease of 0.065 (SE = 0.0032) in the z-score for height (approximately 0.6 cm). Again using pedigree and F_ROHLD_ data from three separate population samples, this gave a much higher estimate of a reduction in height of between 2.8 and 3.3 cm in the offspring of first cousins compared with the offspring of unrelated parents (based on F_ROHLD_ differences of 2.8, 3.3 and 2.9 in the offspring of first cousins compared with the offspring of unrelated individuals in the MICROS, ORCADES and Irish data sets respectively).

The third analysis estimated the association between height and F_hom_, adjusting for age and sex (Figure S2). Again, there was evidence of a very strongly significant inverse association between F_hom_ and height (*p* = 1.10×10^−83^). The direction of effect was consistent for fourteen sample sets, significantly so for seven of these, and not significantly different from zero but of opposite sign in the final seven studies. A 1% increase in F_hom_ was associated with a decrease of 0.11 (SE = 0.0057) in the z-score for height (approximately 1 cm). Again using pedigree and F_hom_ data from three separate population samples, this gave an estimate of a reduction in height of between 2.7 and 3.3 cm in the offspring of first cousins compared with the offspring of unrelated people, identical to the estimate obtained using F_ROHLD_ (based on F_hom_ differences of 2.7, 3.3 and 2.7 in the offspring of first cousins compared with the offspring of unrelated individuals in the MICROS, ORCADES and Irish data sets respectively).

We explored whether the signal observed in the F_hom_ analysis was driven by homozygous genotypes located in long ROH, or from the more common, homozygous genotypes resulting from the chance inheritance of identical shorter haplotypes from both parents. This analysis estimated the association between height and F_hom_, adjusted for age, sex and F_ROH_. Again, a significant association was observed, but both the magnitude and the significance of the effect were reduced compared to the previous analysis (Table 2), suggesting that most, but not all, of the signal was coming from long ROH.

Although these results were highly significant, there was also a high degree of heterogeneity across population samples. Some further analyses were performed to explore the source of this heterogeneity. Three of the twenty-one study samples (Carlantino [INGI-CARL], Lothian Birth Cohort 1936 [LBC1936] and Val Borbera [INGI-VB]) consistently showed a (non-significant) positive association between genome-wide homozygosity and height. In the LBC1936 and INGI-VB cohorts, the parameter estimate was positive for all three measures. In INGI-CARL, the parameter estimate was positive for F_ROH_ and F_ROHLD_; however, the maximum likelihood method used to find the parameter estimate failed to converge for the F_hom_ analysis. Excluding these three cohorts from the F_ROHLD_ meta-analysis reduced heterogeneity considerably, whilst not eliminating it completely (p-value for heterogeneity = 0.01).

Removing these cohorts only slightly reduced heterogeneity in the F_hom_ (p-value for heterogeneity = 6.6×10^−16^) and F_ROH_ meta-analyses (p-value for heterogeneity = 1.3×10^−16^). For both these measures, other outliers also contributed to the heterogeneity. In the case of F_ROH_ the Rotterdam Study (RS) showed a non-significant positive association with height. Four additional cohorts showed a non-significant positive association between F_hom_ and height (EGCUT, CROATIA-Korčula, Queensland Institute of Medical Research [QIMR] and RS).

To summarise, these results provide evidence of a highly significant inverse association between genome-wide homozygosity and height, regardless of which homozygosity estimate was used. The weakest result was for F_ROH_. The effect estimate for this analysis was lower than those for the other 2 homozygosity measures. The most heterogeneous result was for F_hom_. The F_hom_ analysis was similar to F_ROHLD_ in terms of effect size and significance; however, when F_ROH_ was included in the F_hom_ model, although the association remained significant, the effect size fell, the p-value increased and heterogeneity increased. This suggests that the effect was being driven mainly by longer ROH which are more effectively captured by F_ROHLD_. It is important not to overstate this, however: even after controlling for F_ROH_, there is a significant, although highly heterogeneous inverse association between F_hom_ and height, which suggests that a signal is also coming from homozygous genotypes that are not found in the long ROH characteristic of parental relatedness (Table 2). Furthermore, no correlation was observed between sample mean F_ROHLD_ and effect size (r = 0.03). Correlation between these two measures would be expected if the observed effect was entirely attributable to parental relatedness of recent origin. Nevertheless, the most significant and least heterogeneous result was seen with F_ROHLD._ Furthermore, a moderate negative correlation was observed between average F_ROHLD_ and the standard error of the effect estimate (r = −0.4), suggesting that the higher the level of parental relatedness present in the sample, the greater the precision of the effect estimate. This is because mean F_ROHLD_ is related to its standard deviation (higher mean, higher variance) and it is the variance in F_ROHLD_ that determines the standard error of the estimate of the regression coefficient (i.e. higher variance, lower standard error). For these reasons, it was decided to use F_ROHLD_ in further analyses to explore possible confounding factors.

All analyses were adjusted for age but, because the mean age of most of the population samples in this study was over 50 years at the time of genotyping, it was important to undertake additional checks to ensure that the observed effect was not confounded by the effects of osteoporotic, age-related shrinking. We used the Northern Finland Birth Cohort 1966 (NFBC1966), where all subjects were under 40 at the time of measurement. In this cohort, there was a significant inverse association (*p* = 0.002) between F_ROHLD_ and height, with a 1% increase in F_ROHLD_ associated with a decrease of 0.13 in the z-score for height (95% confidence interval −0.16, −0.10). This is equivalent to a reduction in height of 5.3 cm (95% confidence interval −4.1, −6.6) in the offspring of first cousins compared with the offspring of unrelated parents, a stronger effect than observed in the meta-analysis of the full sample. We also repeated the F_ROHLD_ analysis for a subset of individuals aged under 40 years of age (15 cohorts, n = 9909) and the relationship remained significant, although the effect size was much smaller (1% increase in F_ROHLD_ associated with a decrease of 0.009 in the z-score for height (95% confidence interval −0.013, −0.0049; p = 2.15×10^−5^). This is equivalent to a reduction in height of 0.4 cm (95% confidence interval −0.2, −0.5) in the offspring of first cousins compared with the offspring of unrelated parents.

**Figure 3:**
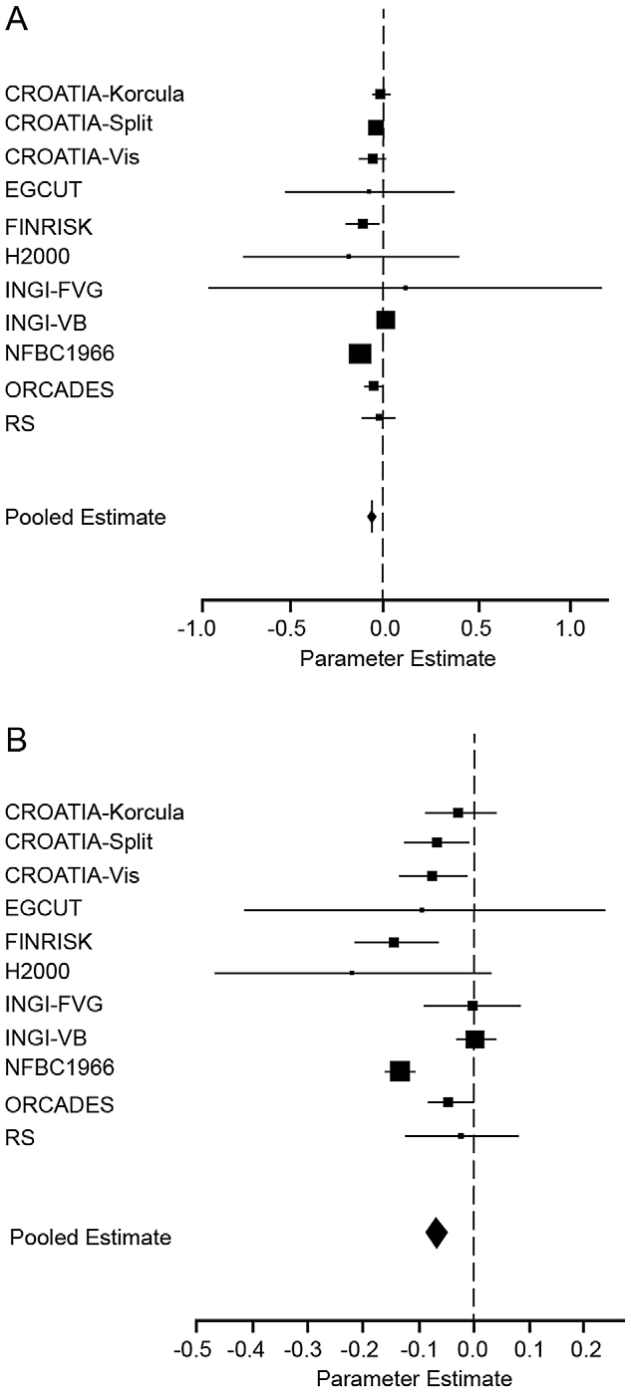
Forest plot of the effect of F_ROHLD_ on height, adjusted for educational attainment. Results of a meta-analysis of the association between F_ROHLD_ and height are shown for the eleven population samples which collected data on educational attainment. (A) shows the model adjusted for age, sex and educational attainment in all samples and additionally for genomic kinship in samples with pairs of related individuals (CROATIA-Korčula, CROATIA-Split, CROATIA-Vis, FINRISK, H2000, INGI-FVG, INGI-VB NFBC1966 and ORCADES). Effect sizes in z-score units (with 95% confidence intervals) are: CROATIA-Korčula = −0.02 (−0.07, 0.04); CROATIA-Split = −0.05 (−0.08, −0.01); CROATIA-Vis = −0.06 (−0.1, 0.02); EGCUT = −0.08 (−0.5, 0.4); FINRISK = −0.1 (−0.2, −0.03); H2000 = −0.2 (−0.8, 0.4); INGI-FVG = 0.1 (−1.0, 1.2); INGI-VB = 0.009 (−0.02, 0.04); NFBC1966 = −0.1 (−0.2, −0.1); ORCADES = −0.06 (−0.1, −0.007); RS = −0.02 (−0.1, 0.08). (B) shows the model adjusted for age and sex in all samples and additionally for genomic kinship in samples with pairs of related individuals (CROATIA-Korčula, CROATIA-Split, CROATIA-Vis, FINRISK, H2000, INGI-FVG, INGI-VB, NFBC1966 and ORCADES). Effect sizes and 95% confidence intervals are as in Figure 2. The plots show estimated effect sizes (solid squares) for each population, with 95% confidence intervals (horizontal lines). Each sample estimate is weighted by the inverse of the squared standard error of the regression coefficient, so that the smaller the standard error of the study, the greater the contribution it makes to the pooled regression coefficient. The area of the solid squares is proportional to the weighting given to each study in the meta-analysis.

The final stage in this analysis was to investigate possible confounding by socio-economic status (SES) of the observed association between genome-wide homozygosity and reduced height. Four of the 21 cohorts (Erasmus Rucphen Family Study [ERF], MICROS, NSPHS and QIMR) did not collect data on SES and so were excluded from further analyses. SOCCS estimated SES using a composite measure of deprivation based on residential address; however, because this was an area- rather than an individual-level estimate and because only one other cohort (ORCADES) used this measure, SOCCS was also excluded from analyses of SES. Eleven cohorts recorded an ordinal measure of educational attainment (CROATIA-Korčula, CROATIA-Split, CROATIA-Vis, EGCUT, the National FINRISK Study [FINRISK], the Health2000 Survey [H2000], FVG-Genetic Park [INGI-FVG], INGI-VB, NFBC1966, ORCADES and RS). Seven cohorts provided an ordinal measure of occupational status (EGCUT, Helsinki Birth Cohort Study [HBCS], INGI-CARL, INGI-FVG, INGI-VB, the Lothian Birth Cohort 1921 [LBC1921], LBC1936 and the Young Finns Study [YFS]); however, the maximum likelihood method used to find the parameter estimate failed to converge for INGI-FVG so this cohort was excluded from the occupational status analysis. We conducted four meta-analyses to investigate whether educational attainment or occupational status confounded the association between genome-wide homozygosity (as measured by F_ROHLD_) and height. First, we analysed the eleven cohorts with educational attainment data available. Two meta-analyses were performed, one adjusting for age, sex, genomic kinship and F_ROHLD_ only and one adjusting for age, sex, genomic kinship, F_ROHLD_ and educational attainment. Results were then compared to assess possible confounding by educational attainment. This process was then repeated for the seven cohorts with data available on occupational status. Results are summarised in Table 3. A forest plot illustrating the results of the educational attainment meta-analyses is shown in Figure 3.

**Table 3:** Meta-analysis assessing potential confounding of SES variables on the association between F_ROHLD_ and height.

Covariates	N samples	N subjects	Effect size	95% Confidence Interval	p-value	p-value (heterogeneity)
**Age, sex, F_ROHLD_**	11	22,430	−0.067	−0.083, −0.051	6.3×10^−17^	1.9×10^−7^
**Age, sex, F_ROHLD_, EA**	11	22,085	−0.068	−0.082, −0.053	5.2×10^−20^	4.9×10^−9^
**Age, sex, F_ROHLD_**	7	10,161	0.0060	−0.020, 0.032	0.65	0.55
**Age, sex, F_ROHLD_, OS**	7	8,459	−0.0063	−0.037, 0.024	0.69	0.073

SES variables are educational attainment (EA) and occupational status (OS).

Inclusion of educational attainment in the model made very little difference to the size, direction and significance of the effect. If anything, inclusion of educational attainment strengthened the association between reduced height and F_ROHLD_, although heterogeneity was also increased. Inclusion of occupational status in the model also made very little difference: in the meta-analysis of the seven cohorts with data on occupational status, no significant association between reduced height and F_ROHLD_ was observed, either with or without the inclusion of occupational status in the model.

## Discussion

This study found evidence for a strongly significant inverse association between genome-wide homozygosity and height (i.e. inbreeding depression) using three alternative estimates of genomic homozygosity, with each method capturing a somewhat different aspect of this phenomenon. Whereas all three measures are strongly correlated, there are also important differences, particularly between F_hom_ and both F_ROH_ measures. For example, whereas the Estonian sample (EGCUT) had the second highest mean value for F_hom_, it had one of the lowest mean values for F_ROHLD_. There are several possible explanations for this. Firstly, it may be suggestive of a small, isolated population deep in the past but with a larger population size and low levels of parental relatedness in recent generations. Secondly, ascertainment bias in the selection of SNPs may also influence these patterns, as markers present in the HumanHap300 panel are more likely to be heterozygous in NW Europeans [37]. Thirdly, it may be that the level of parental relatedness in the sample is lower than that in the population.

The strongest association between genome-wide homozygosity and reduced height was observed using F_ROHLD_, a measure which estimates homozygosity attributable to recent parental relatedness. There is, however, an important caveat: a significant association was also observed between reduced height and F_hom_, controlled for F_ROHLD_, suggesting that homozygous genotypes not located in the long ROH characteristic of recent parental relatedness are also important. We estimated that the increased genome-wide homozygosity that is characteristic of consanguinity results in a reduction of up to 3 cm in the height of the offspring of first cousins compared with the offspring of unrelated parents. Using F_ROHLD_, we then expanded the model to explore possible confounding factors. Firstly, we investigated the possible confounding effects of age-related shrinking. Adult height is the combined effect of growth during childhood and adolescence and loss of height during ageing [11]. There is a powerful age-cohort effect on homozygosity [38] (McQuillan and Wilson unpublished): the rapid pace of urbanisation and population mobility that we have witnessed over the past century has resulted in an observable decrease in homozygosity in younger, compared with older age cohorts. Reduced height is also associated with age, both as a cohort effect reflecting improvements in nutrition and living standards, and because as part of the natural process of ageing, adults lose height as they age due to osteoporotic changes. This process, which is particularly marked in women, may start as young as age 40 [39], with the effects accelerating with age [40]. All analyses were adjusted for age, but as an additional test, we restricted the samples to individuals aged <40. The NFBC1966 sample set provided a further check, as all subjects were aged 31 years at the time of measurement. The inverse association between F_ROHLD_ and height remained in both these analyses, suggesting that confounding as a result of the osteoporotic effects of ageing was not a major factor in these samples. The NFBC1966 analysis also suggests that the relationship between genome-wide homozygosity and height is not confounded by the simultaneous improvements in nutrition and living standards over the last century.

Secondly, we assessed possible confounding by socio-economic status. The association between low childhood SES and reduced adult stature is well established, with the likely mechanism being poor nutrition during childhood [6], although shared genetic factors cannot be excluded. There is no direct evidence on the association between genome-wide homozygosity and SES; however there is a substantial literature on the association between consanguinity, or kin marriage, and SES, albeit not in European populations, where kin marriage is rare. In South and West Asian Muslim populations, where kin marriage is customary, many studies have reported an inverse association between consanguinity and women's educational status [41], although the picture is less clear-cut in men [42]. In a large post-World War Two study of the children of consanguineous parents living in the Japanese cities of Hiroshima and Nagasaki, which used a multi-dimensional SES score, Schull and Neel found a small negative correlation between consanguinity and SES [43]. A later Japanese study also found evidence of confounding by SES, although the direction of the effect was opposite depending on the urban or rural background of the subjects [33]. SES can be estimated in a variety of different ways: the measures available to us here were educational attainment and occupational status. We grouped all the cohorts with ordinal measures of educational attainment together and performed two meta-analyses: one adjusting for age, sex and genomic kinship only and the other adjusting for age, sex, genomic kinship and educational attainment. We compared the two meta-analyses to assess the effect of educational attainment as a possible confounder. We repeated this process for the cohorts with ordinal measures of occupational status. The inclusion of either SES measure in the model made very little difference to the results. We therefore found no strong evidence for confounding by SES, although the limited data available on SES mean that confounding by SES cannot be ruled out entirely.

While we did not have access to raw intensity data with which to call hemizygous deletions, which can masquerade as ROH, two different studies give us confidence that such copy number variation will only have a very minor effect on our results. First, in the ORCADES population, removing ROH which overlapped with deletions resulted in only a 0.3% reduction in the sum length of ROH across the cohort [36]. Second, the median length of these deletions was ∼10 kb in a dataset of >7,000 European-heritage subjects, whereas the median length of ROH in the same studies was ∼2000 kb, showing that the vast majority of deletions will be smaller than the ROH under study here [44]. However, we note that an increased burden of deletions has recently been associated with short stature [45].

Our results are consistent with those of Macgregor and colleagues, who found a significant inverse association between height and both the inbreeding coefficient derived from genealogical data (F_ped_) (p = 0.03; n = 60) and genome-wide homozygosity (p = 0.02; n = 593) in the extreme isolate population of Norfolk Island [34]. The probable reason that they were able to see an effect with such small samples is that they observed much higher levels of parental relatedness than are present in most of the samples used in the present study, therefore the study had greater power to detect an effect. Over one quarter (26%) of their total sample had F_ped_>0, with mean F_ped_ = 0.044. This contrasts with, for example, only 10% of the ORCADES sample having F_ped_>0, with mean F_ped_ = 0.01 using pedigrees of a similar depth (unpublished data). Although comparable pedigree data are not available for all samples, it is probable that, with the possible exception of NSPHS, all the samples in the present study have lower levels of F_ped_ and genome-wide homozygosity and thus lower power to detect an association with height than is the case in the Norfolk Island sample of descendants of the Bounty mutineers. Cultural attitudes to consanguinity are at best ambivalent in Europe, so marriage between first cousins is rare, even in the nine isolated population samples in our consortium, where inflated levels of parental relatedness are predicted simply as a function of population size and endogamy.

The present study's analyses provide strong evidence for an association between genomic homozygosity and reduced height; however, there is also strong evidence of heterogeneity. Although we did not find a significant positive association between F_ROHLD_ and height in any sample, there was a small number of non-significant positive associations and overall there was considerable variation in the magnitude of the observed effects among population samples. One possible explanation for this is that the observed effects are found only in individuals whose parents are closely related (e.g. as first cousins). If this were the case, however, the strongest effects would be observed in the samples with the highest levels of parental relatedness. In fact, we found no correlation between mean sample F_ROHLD_ and effect size. We also found evidence of an association after controlling for parental relatedness, suggesting that homozygous genotypes not resulting from recent parental relatedness also contribute to the observed association. The data do not, then, support the hypothesis that the more inbreeding there is in the sample, the stronger the observed effect. We did, however, find a moderate negative correlation between the mean sample F_ROHLD_ and the SE of the F_ROHLD_ effect estimate, which suggests that the more inbreeding there is in the sample the greater the power to detect an effect and therefore the more precise the estimate of the effect.

One puzzling result of this study was the discrepancy in the results of the meta-analyses of F_ROH_ and F_ROHLD_. The difference in ROH length threshold may contribute to this discrepancy. The 1.5 Mb threshold for F_ROH_ was chosen on the basis of an empirical analysis of several European-heritage populations [36]. All individuals in all samples observed in this study, which also used the Illumina Hap300 SNP array, had ROH<1.5 Mb. ROH longer than this were more common in the offspring of related parents, although still present in most offspring of unrelated parents. With the benefit of hindsight, a longer and thus more stringent ROH length threshold may have been preferable, in terms of differentiating ROH resulting from close parental relatedness originating in recent generations from what might be termed population homogeneity resulting from population isolation deeper in the past. In contrast, the F_ROHLD_ measure does not detect ROH arising from common ancient haplotypes in the population because SNPs in LD are removed before the analysis. Any ROH detected using F_ROHLD_ are the result of parental relatedness of recent origin. For F_ROHLD_ the aim is to maximise the ROH that can be detected by setting a minimum length threshold which is as low as possible. ROH are identified by observing a string of contiguous homozygous genotypes. The greater the number of contiguous homozygous genotypes, the stronger the probability that what is observed is a true ROH (i.e. a segment where the entire stretch of unobserved intervening DNA is also homozygous), rather than just a chance observation. Because of the reduced number of SNPs, and thus reduced SNP density, in the LD-pruned SNP panels used for the F_ROHLD_ analysis, detection of ROH shorter than 1 Mb becomes unreliable: hence 1 Mb was used as the threshold.

The purpose of carrying out this analysis was to investigate possible genome-wide recessive effects on height. These results are important because by showing an association with genome-wide homozygosity rather than specific individual SNPs, we provide evidence that there is a polygenic recessive component to the genetic architecture of height: i.e. that the observed reductions in height associated with genome-wide homozygosity result from the combined effects of many recessive alleles of individually small effect size, located across the genome. The proportion of the phenotypic variance explained by F_ROHLD_ was very variable across cohorts, but the average was 0.4%. Secondly, by demonstrating that the strongest signal comes from the long ROH characteristic of parental relatedness, we provide evidence that the observed effect is primarily the result of rare, rather than common, recessive alleles. Short ROH (measuring up to 2 Mb) are a common feature of all our genomes [36] and their locations are remarkably consistent across different populations, at least within Europe [46]. In contrast, the longer ROH characteristic of parental relatedness are randomly distributed across the genome [36], can be composed of common or rare haplotypes, and as such are predicted to be enriched for rare recessive variants. Our suggestion that it is rare, rather than common, recessive variants that are driving the observed effect is consistent both with theoretical expectations [47] and with empirical data. Two recent studies found evidence that functional regions of the genome (i.e. protein coding regions or regions governing gene expression) are enriched for rare genetic variants. Zhu et al. (2011) conclude that rare, at least moderately harmful, variants constitute the majority of human functional variation [48]. Li et al. (2010) found that non-synonymous coding SNPs were much rarer than synonymous coding SNPs, suggesting that these SNPs have been subject to purifying selection, which in turn suggests that they are deleterious. They found that this pattern was stronger in the X-chromosome than in the autosomes, suggesting that most rare deleterious SNPs are recessive [49].

These findings are also important because, if there is a polygenic, rare, recessive component to the genetic architecture of height, this might also be the case for disease-associated QT of biomedical importance, such as blood pressure and lipid levels. Indeed this is more likely, if these traits are associated with fitness. A high dominance variance has been reported in systolic blood pressure (SBP) and LDL cholesterol in the Hutterites [50]. For this reason, there is a theoretical expectation that these QT will be influenced by genome-wide homozygosity. There have been many empirical studies over the years which have explored this recessive component to the genetic architecture of blood pressure and LDL cholesterol; however until genome-wide scan data became routinely affordable, this could only be investigated indirectly using inbreeding coefficients derived from genealogical data (F_ped_). Such measures are highly error-prone and cannot account for stochastic variation in the inheritance process. Nevertheless, various studies have found evidence of a significant positive association between blood pressure and F_ped_ [51], [52], [53], [54], [55] although other similar studies found no such evidence [56], [57]. One small study by Campbell and colleagues replicated these findings using a genomic measure of homozygosity derived from microsatellite data [32]. Blood pressure in this Croatian island isolate population was significantly (p<0.05) higher in the offspring of consanguineous parents compared with the offspring of unrelated parents. Similarly, there is some evidence of a positive association between total cholesterol and F_ped_ [58] and between low density lipoprotein cholesterol (LDL) and F_ped_ [59] and of a negative association between high density lipoprotein (HDL) and F_ped_ [60], although other studies have come up with more ambiguous results [28], [55]. The study by Campbell and colleagues found significant positive associations between both total cholesterol and LDL cholesterol and homozygosity, using a panel of microsatellite markers. All these, however, were very small studies. The ROHgen consortium is well placed to investigate these questions thoroughly: we have access to large numbers of subjects; we can replicate investigations in a diverse range of European-heritage populations and we have developed a robust methodology applicable to any number of different QT.

## Materials and Methods

### Ethics Statement

Each study had ethical approval for genetic research into the basis of complex traits, approved by the appropriate committees in each country. All participants provided written informed consent. As analyses were performed locally by cohort analysts, no data were shared across national boundaries.

### Study Participants

This meta-analysis combined data from 21 European or European-heritage population samples: The Estonian Genome Centre University of Tartu (EGCUT), the Erasmus Rucphen Family Study (ERF), the National FINRISK Study (FINRISK) (genotyped samples from 1997, 2002 and 2007 study years), the Health 2000 Survey (H2000), the Helsinki Birth Cohort (HBCS), the Lothian Birth Cohort 1921 (LBC1921), the Lothian Birth Cohort 1936 (LBC1936) the Carlantino Project (INGI-CARL), Friuli-Venezia-Giulia-Genetic Park (INGI-FVG), Korčula (CROATIA-Korčula), Micro-Isolates in South Tyrol (MICROS), the Northern Finland 1966 Birth Cohort (NFBC1966), the Northern Sweden Population Health Study (NSPHS), the Orkney Complex Disease Study (ORCADES), Queensland Institute of Medical Research (QIMR), the Rotterdam Study (RS), the Study of Colorectal Cancer in Scotland (SOCCS), Split (CROATIA-Split), Val Borbera (INGI-VB), Vis (CROATIA-Vis) and the Young Finns Study (YFS). Most (n = 16) were population-based samples, 4 were birth cohorts and 1 was a case-control sample. Five study populations were Finnish, 4 were Scottish, 4 were Italian, 3 were Croatian, 2 were Dutch, 1 was Estonian, 1 was Swedish and 1 was Australian of NW European heritage. Most of the samples were drawn from genetically isolated populations or populations with increased homozygosity, such as the Finns. The total number of participants was 35,808. All studies were carried out after the appropriate local ethical approval had been obtained. All participants provided written informed consent. Full sample details are given in Table S1.

### Measurement of Height

In all studies apart from SOCCS, height was measured by trained personnel using a stadiometer. SOCCS participants provided self-reported measurements of height. This was validated by measuring height in a subset of the sample by trained personnel using a stadiometer. There was a high concordance between the two measures.

### Genotyping

All genotyping was performed on the Illumina platform but using four different SNP panels. Seven samples were genotyped using the Illumina HumanHap 300 panel, six using the Illumina HumanHap 370 Duo/Quad panels, five using the Illumina Human 610 Quad panel, one using the Illumina Human 670 Quad panel and one using both the 370 and 610 panels. In order to harmonise the data across samples, SNPs present in the HumanHap 300 panel were extracted and the analysis was conducted using these SNPs only. Quality control procedures were performed on each sample separately, with the minimum requirements as follows. Individuals with more than 5% missing genotypes were excluded. SNPs missing in more than 10% of samples were excluded, as were SNPs failing the Hardy-Weinberg equilibrium test at p<0.0001 and SNPs with minor allele frequency (MAF)<0.01.

### Measures of Genome-Wide Homozygosity

These were detected using the Runs of homozygosity routine in plink [61], [62]. This slides a moving window of 5000 kb (minimum 50 SNPs) across the genome to detect long contiguous runs of homozygous genotypes. An occasional genotyping error occurring in an otherwise unbroken homozygous segment could result in the underestimation of ROH lengths. To address this, the routine allows one heterozygous and five missing calls per window.

#### F_ROH_

ROH were defined as runs of at least 25 consecutive homozygous SNPs spanning at least 1500 kb, with less than a 100 kb gap between adjacent SNPs and a density of SNP coverage within the ROH of no more than 20 kb/SNP. For each individual, an F statistic termed F_ROH_ [36] was derived by summing the lengths of all ROH longer than 1500 kb and expressing this as a percentage of the typed autosomal genome (i.e. the sum of the length of all the autosomes from the first to the last SNP, excluding the centromeres). 1500 kb was chosen as the minimum length of ROH because observational studies in European populations have shown that whereas all individuals have ROH shorter than 1500 kb, ROH longer than this are more likely to be the result of parental relatedness [36]. We have shown previously that this measure is strongly correlated (r = 0.86) with pedigree-derived inbreeding coefficients [36].

#### F_ROHLD_

An alternative approach to deriving an inbreeding coefficient from ROH is to start by pruning the SNP panel of SNPs in strong linkage disequilibrium (LD), in order to remove ROH that are very common due to the high frequency of ancestral haplotypes. SNP panels were pruned using the pairwise option in plink [61]. At each point, it calculates LD between each pair of SNPs in a window of 50 SNPs and removes one of each pair if LD exceeds the user-defined limit (set here at r^2^ = 0.1). ROH parameters were adjusted to reflect the reduced number of SNPs. The minimum number of consecutive homozygous SNPs constituting a ROH was set at 12 (probability of occurring by chance p<0.005 in all samples). The minimum length of ROH was set at 1000 kb, with no more than 250 kb gap between adjacent SNPs and a density of SNP coverage within the ROH of no more than 100 kb/SNP. Individual F_ROHLD_ statistics were then calculated as described above. This approach yields a more stringent estimate of parental relatedness, as it removes all ROH that are there simply because of parental sharing of long haplotypes that are common in the population. ROH consisting of independent SNPs will be of recent origin and will thus be enriched for rarer haplotypes. Again, this is highly correlated with the pedigree-derived inbreeding coefficient (r = 0.82 in a subset of 241 subjects from the ORCADES sample with complete pedigree information available to five ancestral generations).

#### Observed homozygosity (F_hom_)

This is defined as the number of observed homozygous genotypes per individual, expressed as a percentage of the number of non-missing genotypes for that individual. This measure is less strongly correlated with pedigree inbreeding coefficients than the above (r = 0.76 [36]), as it counts all homozygous genotypes and not simply those found in long ROH arising from recent pedigree loops.

### Statistical Analysis

All tests were two sided and a p-value threshold of 0.05 was used. In order to account for differences in mean height among population samples, all height measures are expressed as z-scores. Because genetically isolated populations are characterised by high levels of relatedness between individuals, measures of height are not independent and therefore conventional regression techniques are not appropriate. The CROATIA-Korčula, CROATIA-Split, CROATIA-Vis, ERF, FINRISK, HBCS, H2000, INGI-CARL, INGI-FVG, INGI-VB MICROS, NFBC1966, NSPHS, ORCADES and YFS samples were therefore analysed using a linear mixed polygenic model in GenABEL. This programme maximises the likelihood of the data under the polygenic model with specified covariates. It reports twice the negative maximum likelihood estimates and the inverse of the variance-covariance matrix at the point of maximum likelihood [63], [64], [65]. The z-score for height was analysed with age, sex, genome-wide homozygosity measure and either educational attainment or occupational status fitted as fixed effects. This model also fits a genomic kinship matrix, which estimates pairwise relatedness, derived on the basis of identical by state (IBS) sharing, weighted by allele frequency, so that a pair of individuals sharing a rare allele is estimated to be more closely related than a pair sharing a common allele. All other samples consist of unrelated individuals, so data were analysed in SPSS using simple linear regression, with age, sex, genome-wide homozygosity measure and either educational attainment or occupational status as covariates. Before embarking on analysis of the SOCCS data, the sample was analysed using binary logistic regression to check that height is not associated with colorectal cancer status. There was no association between height and colorectal cancer, so cases and controls were analysed as a single sample.

### Meta-Analysis

Results were combined in a meta-analysis using the inverse variance method to combine effect size estimates from each sample [63]. This weights each sample estimate by the inverse of the squared standard error of the regression coefficient, so that the smaller the standard error of the study, the greater the contribution it makes to the pooled regression coefficient.

### Estimation of the Reduction in Height Resulting from Increased Homozygosity in the Offspring of First Cousins Compared with the Offspring of Unrelated Individuals

In order to standardise across the different population samples in this study, we converted height measurements into z-scores. The results of each meta-analysis report a pooled estimate of the change in this z-score associated with a 1% increase in genomic homozygosity. In order to make this easier to interpret, we express this in the text as the difference in height between the offspring of first cousins and the offspring of unrelated parents. The first step in this analysis was to estimate the difference in observed genomic homozygosity between the offspring of first cousins and the offspring of unrelated parents (a more realistic approach than using the theoretical predictions of F_ped_ = 0.0625 and 0). For each measure of genomic homozygosity we estimated this difference separately in 3 different populations where genealogical and genomic data were available for the reliable identification of the offspring of first cousins. In each population group and for each measure of genomic homozygosity, we estimated the mean difference between the offspring of first cousins and the offspring of unrelated parents. We multiplied this by the effect size estimate from the regression meta-analysis to give a z-score estimate for the reduction in height in the offspring of first cousins compared with the offspring of unrelated individuals. To convert each of these z-scores into cm, we then multiplied them by an estimate of the SD for height across the whole sample, derived by taking the SD for each sample in turn and weighting it by sample size. Two of the three populations used for this analysis were part of the main study (ORCADES and MICROS). The third was a small Irish sample, consisting of members of both settled and traveller communities in Ireland (unpublished data, JF Wilson and GL Cavalleri). We repeated this analysis separately in these three populations, partly because of the very small number of first cousin offspring in any single sample in our study and partly to ensure that the observed difference in homozygosity was not simply an artefact of one particular population sample.

## Supporting Information

Figure S1 Forest plot of the effect of F_ROH_ on height. Results of a meta-analysis of the association between F_ROH_ and height are shown for twenty-one population samples. The model was adjusted for age and sex in all samples. Additionally, it was adjusted for genomic kinship in samples with pairs of related individuals (CROATIA-Korčula, CROATIA-Split, CROATIA-Vis, ERF, FINRISK, HBCS, H2000, INGI-CARL, INGI-FVG, INGI-VB, MICROS, NFBC1966, NSPHS, ORCADES and YFS). The plot shows estimated effect sizes (solid squares) for each population, with 95% confidence intervals (horizontal lines). Each sample estimate is weighted by the inverse of the squared standard error of the regression coefficient, so that the smaller the standard error of the study, the greater the contribution it makes to the pooled regression coefficient. The area of the solid squares is proportional to the weighting given to each study in the meta-analysis. Effect sizes in z-score units (with 95% confidence intervals) are: CROATIA-Korčula = −0.03 (−0.06, −0.003); CROATIA-Split = −0.005 (−0.009, −0.00006); CROATIA-Vis = −0.03 (−0.07, 0.007); EGCUT = −0.04 (−0.3, 0.2); ERF = −0.09 (−0.9, 0.7); FINRISK = −0.09 (−0.2, −0.01); HBCS = −0.05 (−0.2, 0.1); H2000 = −0.16 (−0.2, −0.1); INGI-CARL = 0.01 (−0.03, 0.05); INGI-FVG = −0.03 (−0.05, −0.005); INGI-VB = 0.001 (−0.02, 0.02); LBC1921 = −0.08 (−0.2, 0.03); LBC1936 = 0.07 (−0.1, 0.2); MICROS = −0.05 (−0.02, −0.008); NFBC1966 = −0.08 (−0.1, −0.05); NSPHS = −0.02 (−0.04, −0.008); ORCADES = −0.02 (−0.06, 0.02); QIMR = −0.06 (−0.2, 0.09); RS = 0.003 (−0.06, 0.06); SOCCS = −0.08 (−0.2, 0.05); YFS = −0.05 (−0.1, −0.002).
(TIF)

Figure S2 Forest plot of the effect of F_hom_ on height. Results of a meta-analysis of the association between F_hom_ and height are shown for twenty population samples. For one sample (INGI-CARL) the polygenic model failed to converge. The model was adjusted for age and sex in all samples. Additionally, it was adjusted for genomic kinship in samples with pairs of related individuals (CROATIA-Korčula, CROATIA-Split, CROATIA-Vis, ERF, FINRISK, HBCS, H2000, INGI-FVG, INGI-VB,MICROS, NFBC1966, NSPHS, ORCADES and YFS). The plot shows estimated effect sizes (solid squares) for each population, with 95% confidence intervals (horizontal lines). Each sample estimate is weighted by the inverse of the squared standard error of the regression coefficient, so that the smaller the standard error of the study, the greater the contribution it makes to the pooled regression coefficient. The area of the solid squares is proportional to the weighting given to each study in the meta-analysis. Effect sizes in z-score units (with 95% confidence intervals) are: CROATIA-Korčula = 0.03 (−0.03, 0.09); CROATIA-Split = −0.04 (−0.09, −0.0009); CROATIA-Vis = −0.09 (−0.26, 0.08); EGCUT = 0.002 (−1.9, 1.9); ERF = −0.2 (−0.3, −0.1); FINRISK = −0.1 (−0.2, −0.05); HBCS = −0.09 (−0.4, 0.3); H2000 = −0.2 (−0.4, 0.03); INGI-FVG = −0.27 (−0.33, −0.21); INGI-VB = 0.02 (−0.04, 0.07); LBC1921 = −0.2 (−0.5, 0.05); LBC1936 = 0.02 (−0.2, 0.2); MICROS = −0.07 (−0.1, −0.05); NFBC1966 = −0.1 (−0.3, 0.09); NSPHS = −0.15 (−0.16, −0.13); ORCADES = −0.06 (−0.1, −0.02); QIMR = 0.09 (−0.05, 0.2); RS = 0.007 (−0.07, 0.09); SOCCS = −0.09 (−0.3, 0.2); YFS = −0.1 (−0.2, −0.04).
(TIF)

Table S1 Details of genotyping, QC, data analysis and sample characteristics by cohort.
(XLS)
