# Supplementary figures and images for: Evidence of Inbreeding Depression on Human Height
Source: PLoS Genet. 2012 Jul 19;8(7):e1002655. doi: 10.1371/journal.pgen.1002655 (PMC3400549; doi:10.1371/journal.pgen.1002655)

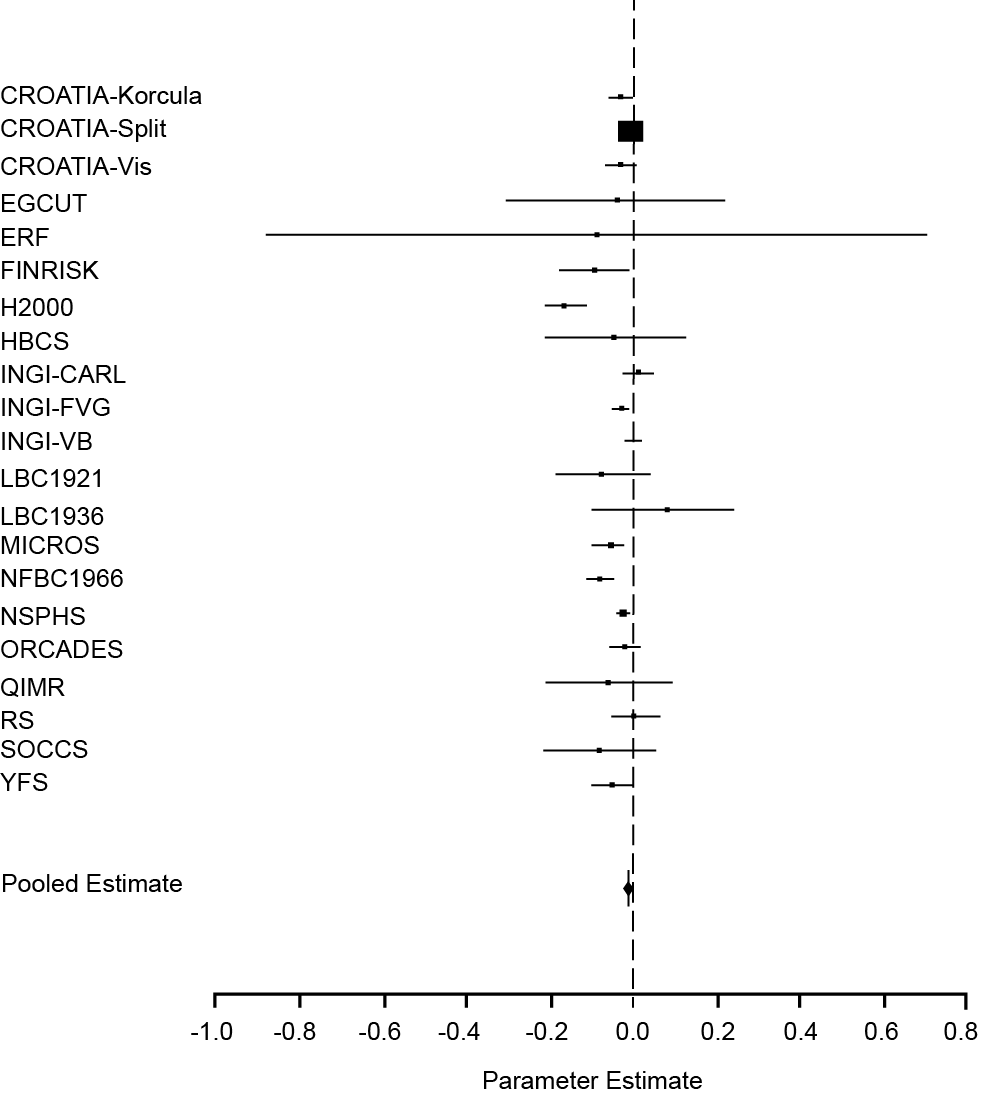

Supplement: Figure S1 — Forest plot of the effect of FROH on height. Results of a meta-analysis of the association between FROH and height are shown for twenty-one population samples. The model was adjusted for age and sex in all samples. Additionally, it was adjusted for genomic kinship in samples with pairs of related individuals (CROATIA-Korčula, CROATIA-Split, CROATIA-Vis, ERF, FINRISK, HBCS, H2000, INGI-CARL, INGI-FVG, INGI-VB, MICROS, NFBC1966, NSPHS, ORCADES and YFS). The plot shows estimated effect sizes (solid squares) for each population, with 95% confidence intervals (horizontal lines). Each sample estimate is weighted by the inverse of the squared standard error of the regression coefficient, so that the smaller the standard error of the study, the greater the contribution it makes to the pooled regression coefficient. The area of the solid squares is proportional to the weighting given to each study in the meta-analysis. Effect sizes in z-score units (with 95% confidence intervals) are: CROATIA-Korčula = −0.03 (−0.06, −0.003); CROATIA-Split = −0.005 (−0.009, −0.00006); CROATIA-Vis = −0.03 (−0.07, 0.007); EGCUT = −0.04 (−0.3, 0.2); ERF = −0.09 (−0.9, 0.7); FINRISK = −0.09 (−0.2, −0.01); HBCS = −0.05 (−0.2, 0.1); H2000 = −0.16 (−0.2, −0.1); INGI-CARL = 0.01 (−0.03, 0.05); INGI-FVG = −0.03 (−0.05, −0.005); INGI-VB = 0.001 (−0.02, 0.02); LBC1921 = −0.08 (−0.2, 0.03); LBC1936 = 0.07 (−0.1, 0.2); MICROS = −0.05 (−0.02, −0.008); NFBC1966 = −0.08 (−0.1, −0.05); NSPHS = −0.02 (−0.04, −0.008); ORCADES = −0.02 (−0.06, 0.02); QIMR = −0.06 (−0.2, 0.09); RS = 0.003 (−0.06, 0.06); SOCCS = −0.08 (−0.2, 0.05); YFS = −0.05 (−0.1, −0.002). (TIF) [file pgen.1002655.s001.tif]

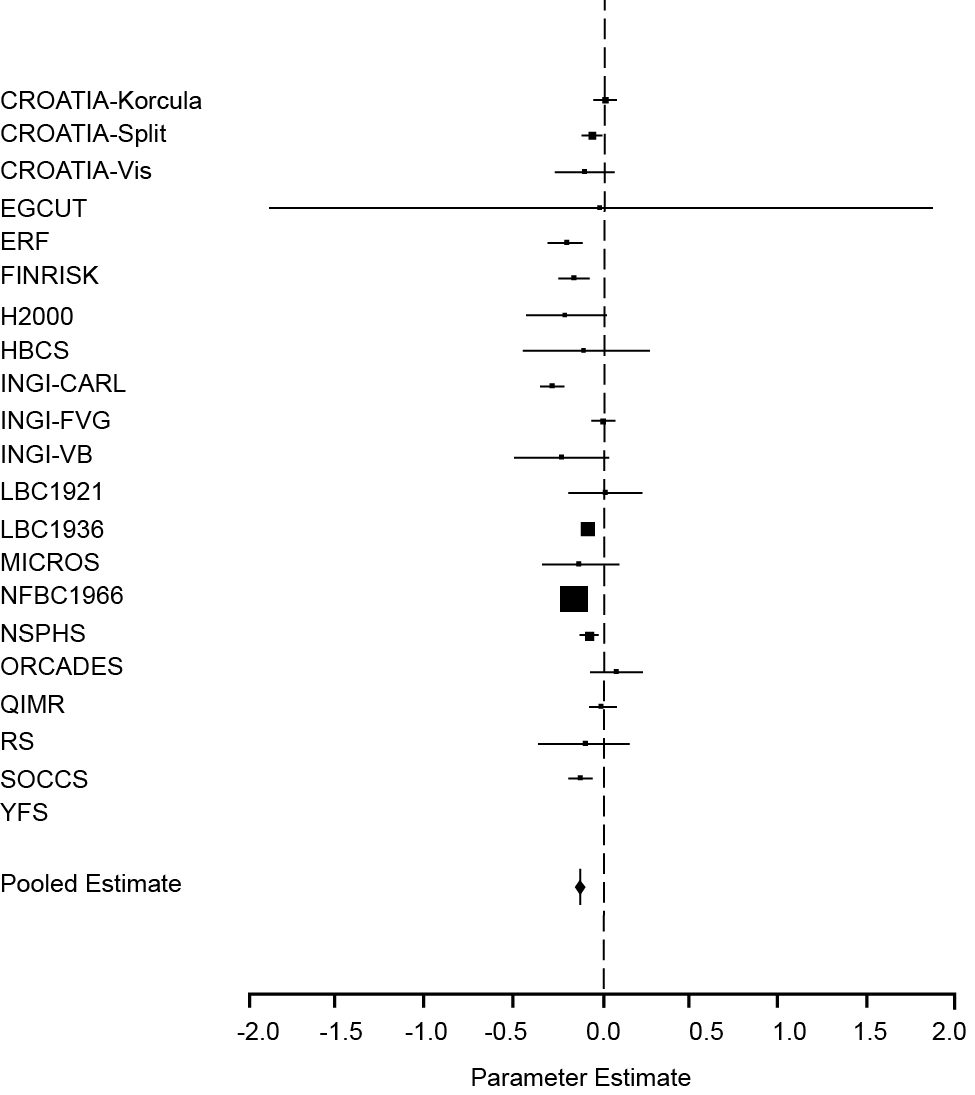

Supplement: Figure S2 — Forest plot of the effect of Fhom on height. Results of a meta-analysis of the association between Fhom and height are shown for twenty population samples. For one sample (INGI-CARL) the polygenic model failed to converge. The model was adjusted for age and sex in all samples. Additionally, it was adjusted for genomic kinship in samples with pairs of related individuals (CROATIA-Korčula, CROATIA-Split, CROATIA-Vis, ERF, FINRISK, HBCS, H2000, INGI-FVG, INGI-VB,MICROS, NFBC1966, NSPHS, ORCADES and YFS). The plot shows estimated effect sizes (solid squares) for each population, with 95% confidence intervals (horizontal lines). Each sample estimate is weighted by the inverse of the squared standard error of the regression coefficient, so that the smaller the standard error of the study, the greater the contribution it makes to the pooled regression coefficient. The area of the solid squares is proportional to the weighting given to each study in the meta-analysis. Effect sizes in z-score units (with 95% confidence intervals) are: CROATIA-Korčula = 0.03 (−0.03, 0.09); CROATIA-Split = −0.04 (−0.09, −0.0009); CROATIA-Vis = −0.09 (−0.26, 0.08); EGCUT = 0.002 (−1.9, 1.9); ERF = −0.2 (−0.3, −0.1); FINRISK = −0.1 (−0.2, −0.05); HBCS = −0.09 (−0.4, 0.3); H2000 = −0.2 (−0.4, 0.03); INGI-FVG = −0.27 (−0.33, −0.21); INGI-VB = 0.02 (−0.04, 0.07); LBC1921 = −0.2 (−0.5, 0.05); LBC1936 = 0.02 (−0.2, 0.2); MICROS = −0.07 (−0.1, −0.05); NFBC1966 = −0.1 (−0.3, 0.09); NSPHS = −0.15 (−0.16, −0.13); ORCADES = −0.06 (−0.1, −0.02); QIMR = 0.09 (−0.05, 0.2); RS = 0.007 (−0.07, 0.09); SOCCS = −0.09 (−0.3, 0.2); YFS = −0.1 (−0.2, −0.04). (TIF) [file pgen.1002655.s002.tif]
